# Expanding Functionality of Commercial Optical Coherence Tomography Systems by Integrating a Custom Endoscope

**DOI:** 10.1371/journal.pone.0139396

**Published:** 2015-09-29

**Authors:** Weston A. Welge, Jennifer K. Barton

**Affiliations:** 1 College of Optical Sciences, The University of Arizona, Tucson, Arizona, United States of America; 2 Department of Biomedical Engineering, The University of Arizona, Tucson, Arizona, United States of America; University Hospital Llandough, UNITED KINGDOM

## Abstract

Optical coherence tomography (OCT) is a useful imaging modality for detecting and monitoring diseases of the gastrointestinal tract and other tubular structures. The non-destructiveness of OCT enables time-serial studies in animal models. While turnkey commercial research OCT systems are plenty, researchers often require custom imaging probes. We describe the integration of a custom endoscope with a commercial swept-source OCT system and generalize this description to any imaging probe and OCT system. A numerical dispersion compensation method is also described. Example images demonstrate that OCT can visualize the mouse colon crypt structure and detect adenoma *in vivo*.

## Introduction

Optical coherence tomography (OCT) is a scanning imaging modality that generates cross-sectional images with high lateral and axial resolution (typically < 15 μm) to depths of up to 2 mm. Originally adopted by the ophthalmic community, OCT has also been used to investigate the gastrointestinal tract [1–5]. At least 40 companies currently produce research or clinical OCT systems [6], including clinical endoscope/catheter-based systems for esophageal (NinePoint Medical) and intravascular (St. Jude Medical) imaging.

Arguably the most challenging and expensive components of an OCT system are the light source and the “engine” responsible for system control, data acquisition, and data processing. Researchers who wish to use OCT may choose to purchase a commercial OCT system that provides the desired imaging speed, axial resolution (lateral resolution is determined by the probe optics), and imaging depth. Few companies produce endoscopic imaging probes for research applications, but several custom probe designs have been published in the literature. Probes can be broadly grouped by their design architecture, such as gradient index lens [7, 8] and fiber [9, 10], traditional lenses [11], ball lens [12], lens generated by melting photonic crystal fiber using the electric arc from a fiber splicer [13], injection of index-matching fluid to improve image quality [14], and lens-free [15]. The best choice of probe architecture (including diameter, flexibility, scanning method, and optical characteristics) depends on the application, thus more variation in probe design is needed than in OCT engine configuration. Compared to the light source and engine, probes are typically easier and less expensive to construct.

**Fig 1:**
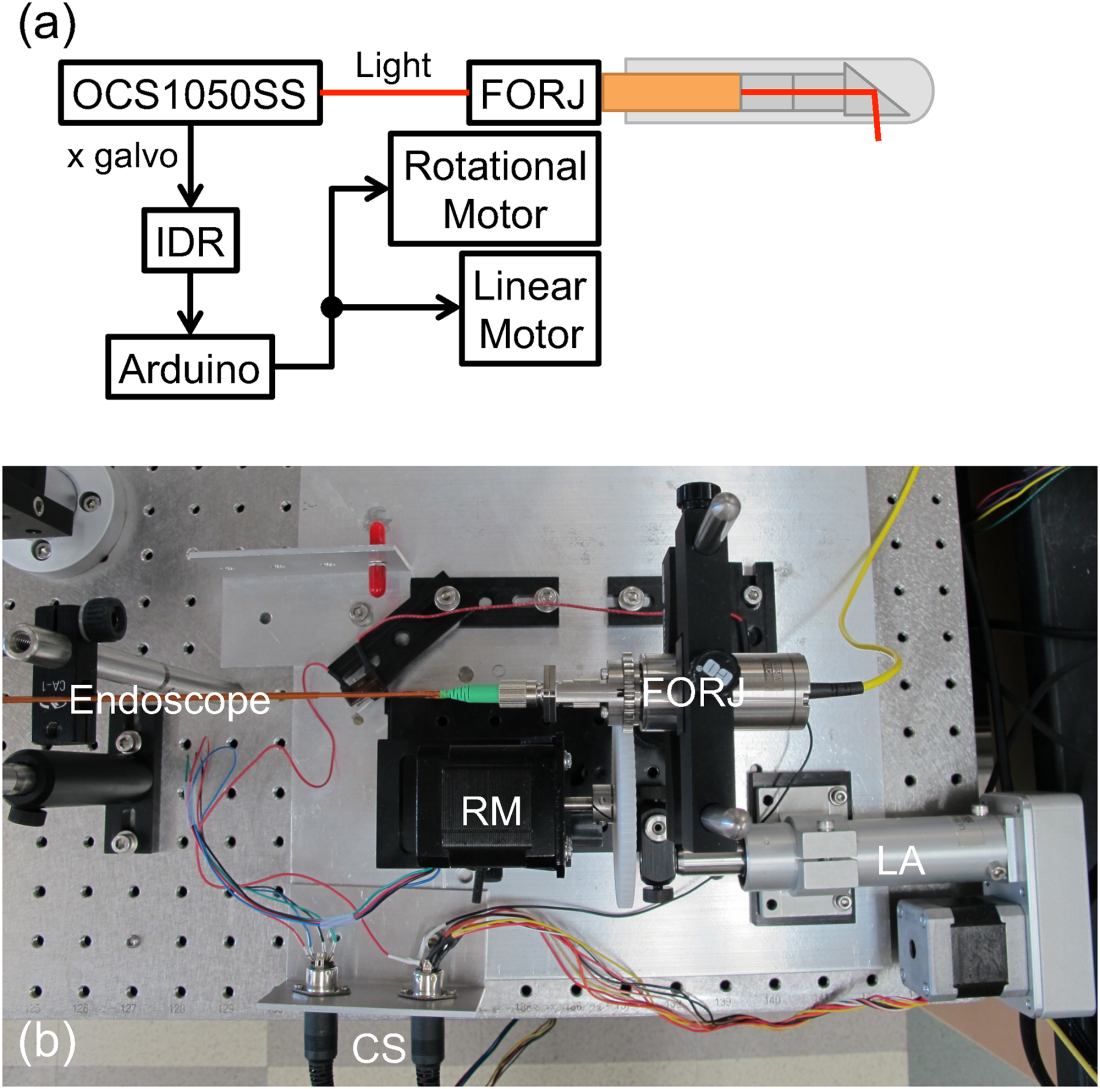
System diagram. (a) Thorlabs OCS1050SS swept-source OCT system is treated as a black box. The fiber optic rotary joint (FORJ) is connected to the OCS1050SS light source via a fiber optic patch cable. The electronic *x*-galvanometer scanner control signal from the OCS1050SS is connected to a custom inverting differentiator and rectifier (IDR) circuit that converts the sawtooth input wave to a square wave with a high signal during the period of data acquisition along the fast *x*-axis of the raster scan pattern. The IDR output is connected to an Arduino Uno that detects the rising edge and outputs a short digital pulse to signal the motor drivers to begin movement of the rotational and linear motors. The endoscope is connected directly to the FORJ. The rotational motor is connected to the FORJ through meshed gears. The rotational motor, FORJ, and endoscope are mounted on a linear translation stage that is controlled by the linear motor. The fast rotational motion and slow linear motion result in a helical scanning geometry. (b) A photograph of the sample arm, including the motors and translation stage. The rotational motor (RM) spins the fiber optic rotary joint (FORJ) and both are mounted on a linear translation stage that is moved by the linear actuator (LA). Motor drivers send control signals (CS) to the control the motors.

This article describes the integration of a lab-built gradient index lens-based endoscope [16] with a commercial swept-source OCT system (OCS1050SS, Thorlabs, Newton, NJ, USA), which has a central wavelength of 1040 nm and spectral bandwidth of 80 nm. The overall system diagram is shown in Fig 1. The swept-source laser in this system produces an axial resolution of 12 μm in air, and the A-scan rate of the system is 16 kHz. The OCT system has an imaging range of 2.0 mm, A/D sample rate of 100 MS/s, and a 3 dB rolloff of 0.47 mm. The system generates fringe data linearly in wavenumber. This central wavelength provides a compromise of resolution and penetration depth in highly scattering tissue. Furthermore, this wavelength is useful for ophthalmic imaging due to fairly low water absorption. The bandwidth and A-scan rate are sufficient for video-rate imaging at good axial resolution. The choice of light source impacts the performance of the OCT system in several ways that are described below.

The details in this article, however, can be generalized to the integration of any custom imaging probe to any commercial OCT system that provides a connection to the sample arm optics and access to scan synchronization signals. When the user is unable to directly control the data acquisition and OCT control software, the system is best viewed as a black box. In other words, the sample arm optics and scanning motion must be designed around the constraints imposed by the OCT system.

Examples of such constraints are the imaging speed, data acquisition scheme, and optical materials. The A-scan rate is generally constant, but the B-scan rate may be variable. If the OCT system allows the user to adjust the physical scan length and/or sampling density, then the total time to acquire a single B-scan will change. The B-scan acquisition time constrains the custom sample arm scan length, velocity, and sampling density.

The data acquisition scheme encompasses the scanning geometry and any computational overhead involved in recording and processing data. For a raster scanning system, data are likely only acquired during one sweep direction of the fast scan axis. If there are brief time periods of no data acquisition between each subsequent B-scan, this constrains or complicates scanning geometries for the custom sample arm. On the other hand, if the commercial system acquires data by scanning the beam in a spiral, then data may be acquired constantly. This provides greater freedom for designing the new sample arm scanning geometry, but the images generated by the system would be distorted if the new sample arm scans in a non-spiral geometry. In general, whenever the scanning geometry changes, the image processing by the OCT system will generate distorted images. To properly display the images, the user must process the image data themselves.

Finally, the optical materials used in the system, such as fibers and lenses, may not be known to the user. This poses two problems. The first is matching the optical path length of the new sample arm to the commercial arm in order to generate OCT images. Building the custom endoscopic sample arm with a matching optical path length may require measuring the physical length of one of the commercial arms (sample arm would most often be easiest) and multiplying by the refractive indices of an appropriate fiber and lenses for the source spectrum. Designing the sample arm with the necessary optical path length may require trial and error. The second problem is dispersion. The broad bandwidth sources used by high-resolution OCT systems are particularly susceptible to the effects of dispersion mismatches between the sample and reference arms. The simplest way to mitigate dispersion is to ensure that the amount of dispersive materials are equal in the sample and reference arms. However, if the reference arm specifications are not completely known, it may be difficult to avoid mismatching the dispersion in the sample arm. A variable-thickness prism pair may be placed in an air gap in the sample arm or reference arm if room is available. The thickness can be adjusted through trial and error until axial-resolution degradation is minimized. However, there may not be space available to physically correct for mismatched dispersion and complete compensation is unlikely. If this is the case, numerical dispersion compensation methods can be applied to fix the axial blurring in post-processing. These methods require access to the raw fringe data.

We describe our method of integrating an endoscope with the OCS1050SS system as a specific example. Integration details are provided below in three parts: the sample arm optics, scanning synchronization, and numerical dispersion compensation. Highly sampled images of normal and adenomatous mouse colon are provided in the Results section. These images demonstrate how a system designed for microscope stage imaging can be modified to accommodate an endoscope capable of resolving colonic crypts and enable the measurement of adenoma size and tumor burden.

## Materials and Methods

### Light source

The light source affects OCT performance in many ways and the ideal light source will depend on the particular application. In OCT, the axial and lateral resolutions are decoupled (the lateral resolution depends on the imaging probe optics). If the source spectrum is approximately Gaussian, then the axial resolution Δ*z* in a medium characterized by refractive index *n* depends on the central wavelength *λ*_0_ and the bandwidth Δ*λ* according to

$$\begin{array}{c} \Delta z=\frac{2\ln(2)}{\pi n}\frac{\lambda_{0}^{2}}{\Delta \lambda }. \end{array}$$ (1)

The result of this computation is sometimes referred to as the coherence length of the source. For a broadband source that simultaneously emits at all wavelengths within its bandwidths (e.g., superluminescent diode), the coherence length and the axial resolution are the same. However, the coherence length of swept-source lasers—which have narrow bandwidths at each instance in time, but sweep over a large bandwidth—is often specified as the instantaneous coherence length and is therefore much larger than the axial resolution. Nevertheless, Eq (1) can still be used to compute the theoretical axial resolution of a swept-source laser for OCT.

The penetration depth of OCT is limited by light scattering and absorption, both of which depend on wavelength. Absorption by chromophores present in biological tissue (primarily oxyhemoglobin, deoxyhemoglobin, and water) varies greatly with wavelength, but is relatively low in the region of 600 nm to 1300 nm, where the absorption coefficient *μ*_*a*_ is approximately 0.1mm^−1^ to 1.0mm^−1^. Scattering monotonically decreases with increasing wavelength and dominates in this spectral region with reduced scattering coefficient $\mu_{s}^{\prime }$ approximately 10mm^−1^ to 100mm^−1^ [17]. When $\mu_{s}^{\prime }\gg \mu_{a}$, the diffusion approximation can be used to accurately estimate the penetration depth. The penetration depth is defined as the distance by which the light intensity drops to 1/*e* of the incident intensity and is defined as

$$\begin{array}{c} \delta =\frac{1}{\sqrt{3\mu_{a}\left(\mu_{a}+\mu_{s}^{\prime }\right)}}. \end{array}$$ (2)

Common commercial swept-source lasers for OCT applications have central wavelengths at or near 850, 1050, 1310, 1550nm [18]. Table 1 shows a comparison of penetration depth and axial resolution at these wavelengths. Reduced scattering coefficient can be estimated as

$${\begin{array}{c} \mu_{s}^{\prime }=a\left(\frac{\lambda }{500}\right) \end{array}}^{-b},$$ (3)

where *λ* is the central wavelength in nanometers, *a* is the value of $\mu_{s}^{\prime }$ at *λ* = 500nm, and *b* is the scattering power [19]. Experimental values for *a* and *b* in bowel tissue are 1.65mm^−1^ and 1.240, respectively [19]. The absorption spectrum is not so easy to estimate at arbitrary wavelengths and the literature is sparse in describing tissue absorption at wavelengths longer than 1000mm. We approximate $\mu_{a}\approx 0.1\mu_{s}^{\prime }$ in the calculations for *δ* in Table 1. The axial resolution assumes a spectral bandwidth of 100nm.

**Table 1:** Comparison of Estimated Penetration Depth and Axial Resolution in Colon Tissue for Four Common OCT Wavelengths.

*λ*_0_ (nm)	*δ* (mm)	Δ*z* (μm)
850	2.04	2.28
1050	2.65	3.48
1310	3.48	5.41
1550	4.29	7.57

*λ*_0_, central wavelength; *δ*, estimated penetration depth; Δ*z*, axial resolution in tissue with index of refraction 1.4 and assuming a constant bandwidth of 100 nm.

Water is a particularly important absorber in biological tissue. Most tissue has a higher concentration of water than of other significant absorbers. At wavelengths shorter than 1100 nm, both oxygenated and deoxygenated blood have higher absorption coefficients than water, but water dominates at higher wavelengths [19]. Water absorption has a local minimum near 1050 nm, making that wavelength an attractive choice for imaging through the eye.

The best choice of light source wavelength depends on the application. Generally, in highly scattering tissues there exists a tradeoff between imaging depth and axial resolution given limited choice of source bandwidths. Other considerations include that standard optical fibers exhibit zero dispersion at 1310 nm, which may simplify dispersion correction, and the broad availability of fiber optical components optimized for 1310 nm and 1550 nm. For our application, the satisfactory penetration depth and high resolution available at 1050 nm led to the choice of this wavelength source.

### Sample arm optics

**Fig 2:**
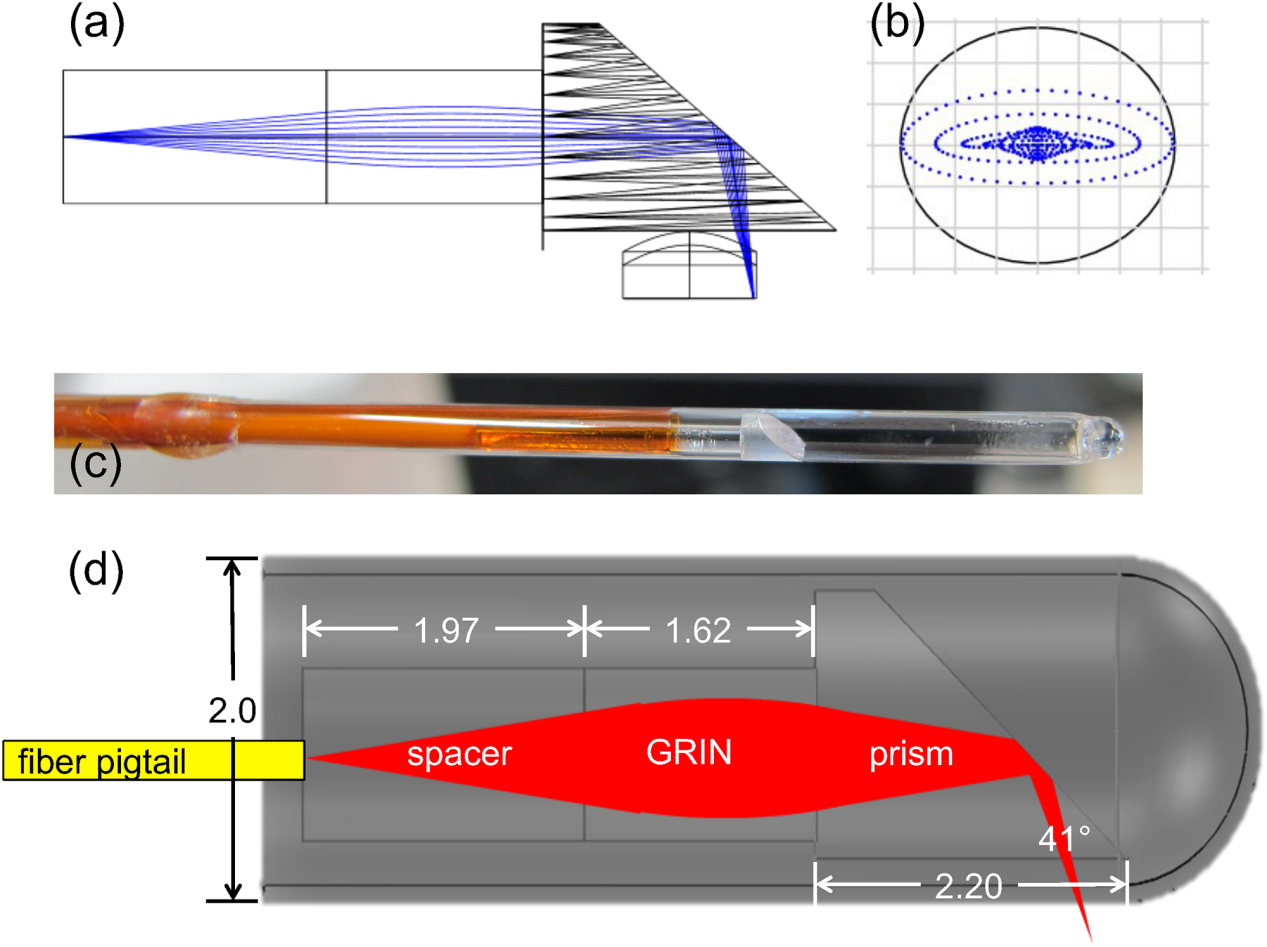
Endoscope. (a) Optical layout showing rays traced through the glass spacer, gradient-index lens, 41° prism, glass window, and tissue. (b) Spot diagram at beam focus. The cylindrical glass window induces astigmatism, but the traced spot is still smaller than the diffraction limited spot radius of 6.14 μm (denoted by the black circle). (c) Photograph of the distal portion of the endoscope. (d) Diagram showing dimensions in mm. Both the spacer and GRIN lens have a diameter of 1.0 mm. The prism is cylindrical with a rectangular exit face. The radius of the circular entrance face is 0.85mm with the square exit face cut a distance of 0.7mm from the center of the circular entrance face. The wall thickness of the glass envelope is 100 μm and a length of about 35 mm. The GRIN lens is characterized by a numerical aperture of 0.5 and a pitch of 1.4.

The sample arm replaced the tabletop microscope setup standard with the Thorlabs OCT system. The design was previously reported [16], and an overview is given here. The Zemax (Zemax, Kirkland, WA, USA) optical design and spot diagram are shown in Fig 2. The new optics are connected to the fiber optic output connector on the OCT instrument. The optics consist of a fiber optic patch cable, fiber optic rotary joint (FORJ), and a 2mm-diameter, side-viewing endoscope in a commonly used configuration [7]. The endoscope consists of a single-mode optical fiber (HI 1060, Corning, Corning, NY, USA) cemented to a BK7 glass spacer, followed by a gradient-index lens (0.14 pitch, 0.5 lens NA, Grintech, GmbH, Jena, Germany) and a 41° rod prism (Photop Technologies, Sunnyvale, CA, USA), all encapsulated with a glass cylindrical window (custom, University of Arizona Glass Shop) attached to a polyimide tube (MicroLumen, Oldsmar, FL, USA). The length of the spacer (1.97mm) and the pitch of the gradient-index lens were chosen to achieve a numerical aperture (NA) to match the NA of the fiber at 0.14. The endoscope provides a lateral resolution of 6 μm, with the focus of the beam located approximately 250 μm outside the window. The total length of the endoscope is 295 mm. The FORJ (Princetel, Hamilton, NJ, USA) enables free rotation of the endoscope. The length of the fiber optic patch cable was chosen so that the optical path length of the custom probe was the same as the original sample arm (and thus would match the OCT system reference arm length). The OCS1050SS reference arm length can be adjusted by about 2 cm. The desired length of the new sample arm was estimated by measuring the physical length of the commercial probe fiber and estimating the path through the probe. Starting from this estimate and the known optical path lengths through the FORJ and endoscope, the correct patch cable length was determined through trial and error. An image will be generated with the OCT system once the optical path length of the sample arm and reference arm match within the coherence length of the source.

The endoscope collects data in a helical scanning geometry using a fast rotational stepper motor (Lin Engineering, Morgan Hill, CA, USA) and a slow linear actuator (Ultra Motion, Cutchogue, NY, USA), each controlled by a motor driver (3540i, Applied Motion Products, Watsonville, CA, USA). The beam always passes through the cylindrical glass window during data collection. The window and polyimide sheath are held stationary while the internal optics move independently. A spur gear attached to the rotational stepper motor meshes with the gear teeth on the FORJ for rotational motion. The FORJ, rotational motor, and internal endoscope optics are all mounted on a linear translation stage connected to the linear actuator for longitudinal motion. A popular alternative that avoids the use of a FORJ is to attach the prism to a rotational micromotor at the distal tip of the endoscope [20]. A downside of this approach is that the micromotor wires inherently obstruct part of the field of view. Another design scans using a piezoelectric transducer [21], which is relatively complex, but could be made side-viewing by placing a cone mirror at the distal tip of the endoscope.

### Scanning synchronization

The scanning motion of the sample arm can be synchronized to the commercial OCT scanning signals to avoid changing the data acquisition rate and image processing. In this way, the sample arm appears “unchanged” from the perspective of the OCT system. Many commercial systems steer the imaging beam in the sample arm with a scanning *x*-*y* galvanometer mirror system. The OCS1050SS sample arm uses a raster scan pattern. Thus to synchronize the new helically scanning endoscope, the rotational motor was synchronized to the fast *x*-axis of the galvanometer system such that one full rotation corresponded to the period of data acquisition of one sweep of the original *x*-galvanometer mirror.

The speed of the linear motor controls the pitch of the helix. If this linear motion is slow, the endoscope linear motor can move continuously without significant image distortion, thus restricting the burden of synchronization to the rotational motion. Therefore, the slow *y*-galvanometer signal was not used. Linear motion speed and time were empirically adjusted such that the motor moved the desired distance within the total 3D image acquisition time of the OCT instrument.

**Fig 3:**
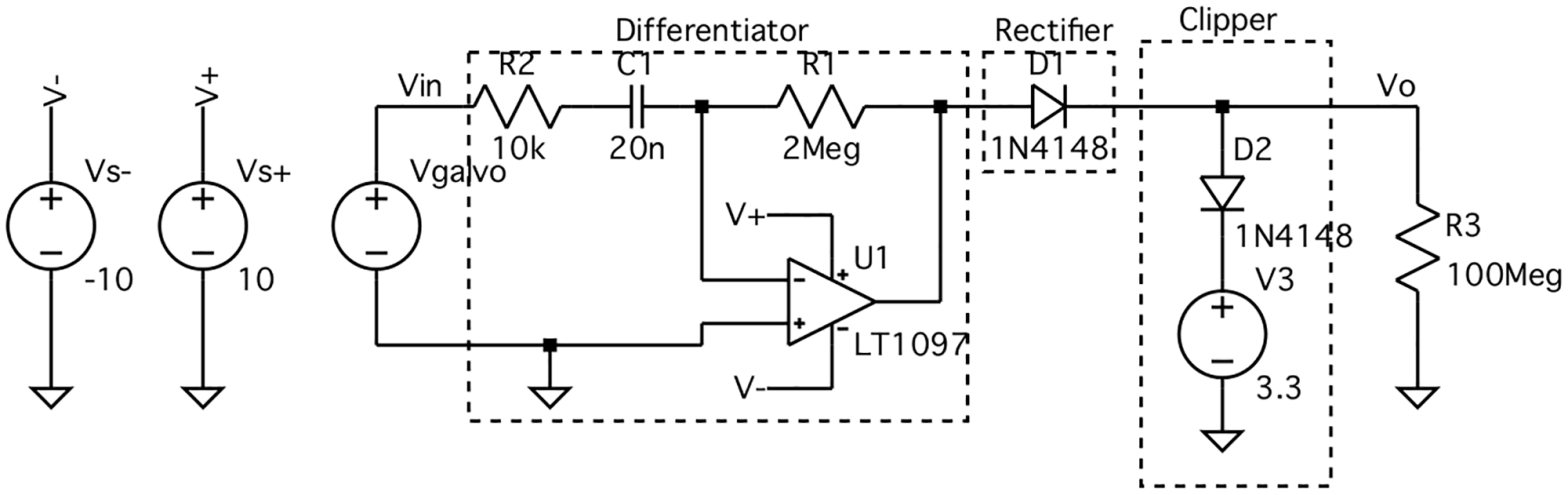
Inverting differentiator and rectifier circuit schematic. This circuit converts the *x*-galvanometer waveform (V_galvo_) from a sawtooth wave with a peak-to-peak voltage of ±10V to a square wave that is 4V during the slow sweep of the *x*-galvanometer mirror and 0V otherwise. The V_galvo_ waveform is characterized by three parts. Data are acquired during the low-magnitude negative slope portion of the wave. The rapid return of the *x*-galvanometer mirror occurs during the high-magnitude positive slope portion. In between these two sweeps, the raw OCT fringe data is saved to the PC and the V_galvo_ waveform is kept constant (zero slope). The purpose of this circuit is to detect the beginning of the negative slope portion of the waveform and communicate this to an Arduino Uno, which can safely read input voltages from 0V to 5V. Resistor R2 attenuates high-frequency ringing in the Differentiator. The Rectifier diode holds the negative portion of the Differentiator output at 0V. The optional Clipper prevents the output V_o_ from exceeding 4.3V (the sum of the bias voltage V3 and the forward voltage drop of D2). The input impedance of the Arduino I/O pins is represented by R3.

There may be a difference between the signal waveform type and amplitude generated by the commercial OCT system (an analog ± 10V sawtooth in our case), and that needed by the endoscope motor drivers (a digital TTL signal). If so, additional electronics will be needed to synchronize the motors and OCT control signals. We used an analog inverting differentiator and rectifier circuit and a microcontroller (Arduino Uno) to generate a short, 5V digital pulse signal immediately after the *x*-galvanometer control signal indicated the beginning of a sweep (by beginning a ramp from 10V to −10V). The inverting differentiator and rectifier circuit schematic is shown in Fig 3. The inverting differentiator output is given by

$$\begin{array}{c} v_{\text{out}}\left(t\right)=-RC\frac{dv_{\text{in}}\left(t\right)}{dt}, \end{array}$$ (4)

where *R* = 2 MΩ, *C* = 20 nF, *v*_in_(*t*) = *V*_galvo_, and *v*_out_(*t*) is the output of the LT1097 operational amplifier. The rectifier holds any negative output voltage from the inverting differentiator at 0V. The gain and rectifier ensure that the circuit output range is within the 0V to 5V range supported by the Arduino input/output pins.

**Fig 4:**
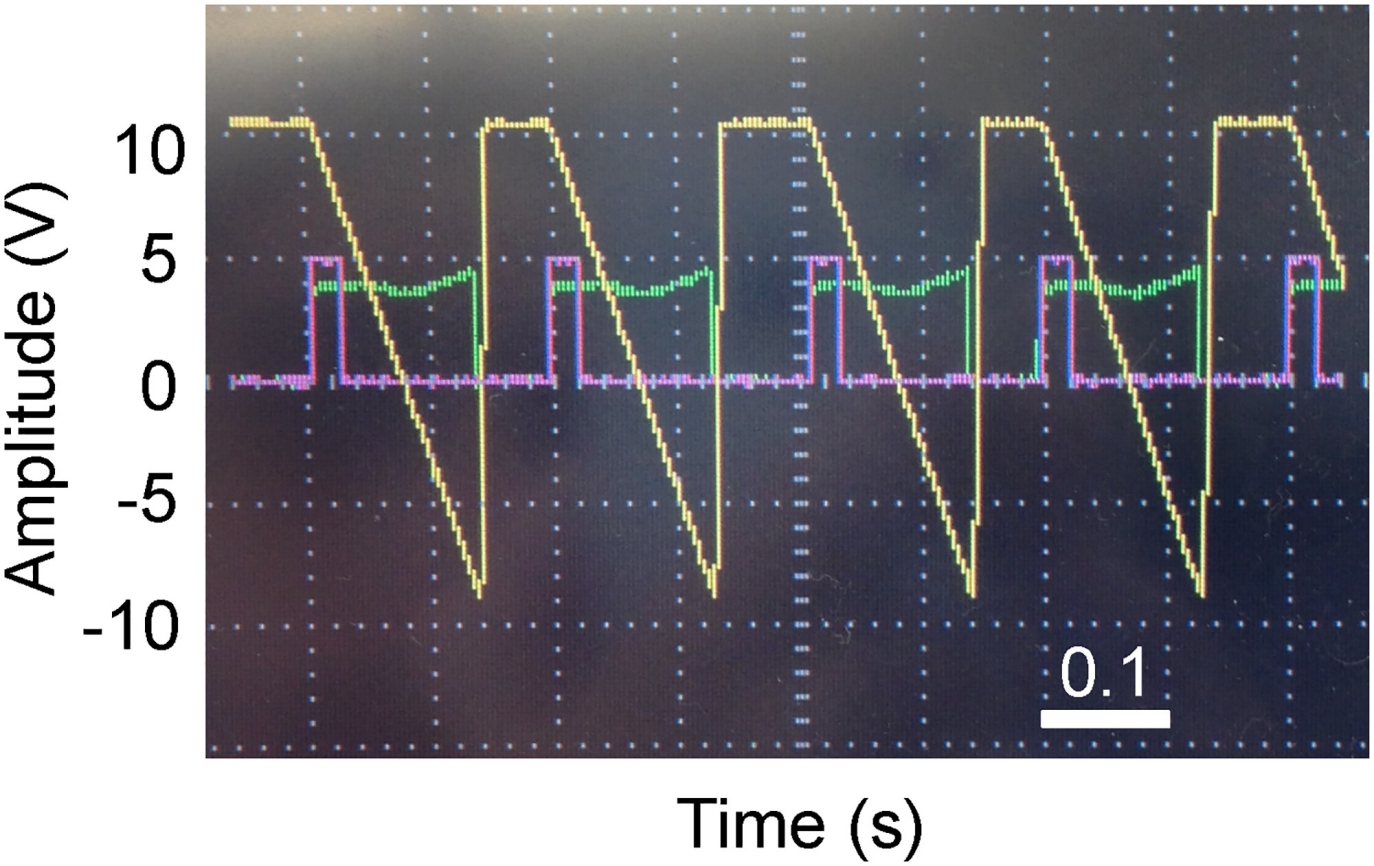
Oscilloscope traces of motor control signals. A few cycles of the electronic signals used in motor synchronization are displayed. The *x*-galvanometer control signal is yellow, the output of the inverting differentiator and rectifier circuit is green, and the Arduino output that serves as the digital pulse to trigger the motors is purple.

The Arduino was programmed to output a 25 ms digital pulse upon detecting a rising edge from the inverting differentiator and rectifier circuit. Fig 4 shows an oscilloscope trace showing the *g*-galvanometer signal, inverting differentiator and rectifier output, and Arduino output. The inverting differentiator and rectifier output was connected to an external interrupt pin on the Arduino. A rising-edge triggers an interrupt service routine that simply sets an output pin to 5V, which is connected to the input of the motor drivers. By inspection of the generated assembly code, the total delay between the triggering of the interrupt and setting the output pin high was 4 μs. The duration of each rotation (B-scan) was about 140 ms, so this delay results in a loss of 0.002% of data. This is minor in our case, but the error increases with high B-scan rates. As such, the delay introduced by any control electronics should be carefully checked to ensure that it does not result in a significant loss of data.

In addition to motion synchronization, the velocity and acceleration of the motors controlling the endoscope can be chosen to minimize image distortion and produce the desired sampling density. In a raster scan, data are not collected during the rapid return sweep of the fast *x*-axis. Therefore, each B-scan will be aligned with respect to one another when the rotational motion of the endoscope comes to a complete stop after each rotation. The angles during which the endoscope experiences rotational acceleration and deceleration will be oversampled. This distortion can be fixed by resampling the data if information about the position or instantaneous rotational velocity are known. This information can be collected using, for example, a closed-loop encoder or Hall effect sensor with the rotational motor. Care must be taken that the entire 360° scan is accomplished within one *x*-axis data acquisition sweep (B-scan). In the OCS1050SS system, as in most swept-source OCT systems, the A-scan rate is constant. The lateral sampling density is therefore determined by the rotational velocity.

For very fast systems, starting and stopping the rotational motion for each B-scan may be unfeasible. An alternate rotational scanning method would be to continuously rotate the endoscope at a constant rotational velocity. Each B-scan would be rotationally misaligned from one another based on the time duration where data are not collected between B-scans. If this time is constant, then a constant circular shift can be applied to the raw fringe data to align the B-scans. If this time varies, as was the case with the OCS1050SS, then each B-scan could be aligned after determining the rotational offset by measuring the rotational orientation of the endoscope with a Hall effect sensor or by computing the maximum correlation of adjacent B-scans.

### Numerical dispersion compensation

Dispersion is a phenomenon by which the speed of light in a medium depends on the wavelength. If the total group index of all media in the sample and reference arms mismatch, the axial resolution will be degraded. Dispersion is often corrected physically by matching media in the sample and reference arms, and/or by placing compensating optics such as a variable-thickness prism pair in the optical path of one of the arms. Complete physical compensation may be impossible due to space and access constraints of commercial systems. In such cases, numerical dispersion compensation methods can be applied to correct axial resolution degradation.

Several methods exist for numerical dispersion correction in OCT [22–25]. The simple method below, adapted from [26], requires that the fringe data be uniformly sampled by wavenumber prior to correction. This method is computationally simpler than the methods described in [22–25], but is only appropriate for samples that introduce minimal dispersion (i.e. this method is appropriate for correcting dispersion introduced by the OCT system). The measured fringe signal in frequency-domain OCT contains two DC terms corresponding to the reflectance of the sample and reference mirror plus a modulation term that is proportional to

cos(k(ω)z+ϕdisp(k))=Re{exp[i(k(ω)z+ϕdisp(k)])}, (5)

where *k*(*ω*) = *ωn*(*ω*)/*c* is the wavenumber as a function of angular frequency *ω*, *z* is the depth in the sample measured relative to the location of zero optical path difference, and *ϕ*_disp_(*k*) is the phase due to dispersion as a function of wavenumber. Recognizing that, in the absence of dispersion, the modulation frequency is linearly proportional to the optical path difference (*k*(*ω*)*z* = *ωOPD*(*ω*)/*c*), a line is fit to the measured phase as a function of *k* and subtracted from the phase, effectively isolating *ϕ*_disp_(*k*), which is nonlinear.

The method we used in practice is described in Alg. 1. The system dispersion was determined from a sequence of 2000 A-scans collected at the same location on a mirror. The raw fringes were averaged together to obtain a smooth fringe measurement. The instantaneous phase (the argument of the modulation term) was extracted by taking the angle of the Hilbert transform of the averaged fringe signal. A linear fit was computed using iteratively reweighted least squares with bisquare weighting (robustfit function in Matlab (2014a, Mathworks, Natick, MA, USA)). The system dispersion *ϕ*_disp_(*k*) is the difference of the instantaneous phase and the linear fit. DC signal removal was performed on sample images by subtracting the source spectrum from the sample fringes. The spectrum was estimated by averaging all fringes in a B-scan. Finally, the degradation due to dispersion was corrected by multiplying the sample fringe data after DC removal by exp(−*iϕ*_disp_). Because the swept source in the OCS1050SS has asymmetric forward and reverse sweep spectra, the entire dispersion compensation algorithm was applied independently to the fringes corresponding to the even and odd A-scans.

**Algorithm 1:** Numerical dispersion compensation.

**Require**: $f_{k,i}^{\left(\text{M}\right)}$ is the fringe data corresponding to the *k*th pixel of the *i*th A-scan taken from a mirror. Each mirror A-scan is collected at the same position. $f_{k,i}^{\left(\text{S}\right)}$ is the fringe data from the tissue sample. All fringes are linearly sampled by wavenumber *k*.		
1:	**function** DispersionCompensation ($f_{k,i}^{\left(\text{M}\right)}$,$f_{k,i}^{\left(\text{S}\right)}$)	
2:	${\bar{f}}_{k}^{\left(\text{M}\right)}\leftarrow \frac{1}{N}\sum_{i=1}^{N}f_{k,i}^{\left(\text{M}\right)}$	▹ ${\bar{f}}_{k}^{\left(\text{M}\right)}\propto \text{cos}(k\cdot OPD+\phi_{\text{disp}}(k))$
3:	**for** *i* = 1 to *N* **do**	▹ Spectral subtraction
4:	$f_{k,i}^{\prime (\text{M})}\leftarrow f_{k,i}^{\left(\text{M}\right)}-{\bar{f}}_{k}^{\left(\text{M}\right)}$	
5:	**end for**	
6:	${\tilde{f}}_{k,i}^{\left(\text{M}\right)}\leftarrow f_{k,i}^{\prime (\text{M})}+iℋ\left\{f_{k,i}^{\prime (\text{M})}\right\}$	▹ ${\tilde{f}}_{k,i}^{\left(\text{M}\right)}$ is the analytic representation of $f_{k,i}^{\prime (\text{M})}$
7:	$\phi_{k,i}^{\left(\text{M}\right)}\leftarrow \text{arg}\left\{{\tilde{f}}_{k,i}^{\left(\text{M}\right)}\right\}$	
8:	${\bar{\phi }}_{k}^{\left(\text{M}\right)}\leftarrow \frac{1}{N}\sum_{i=1}^{N}\phi_{k,i}^{\left(\text{M}\right)}$	
9:	Compute robust linear regression of $\phi_{k}^{\left(\text{M}\right)}$, returning coefficients *β* (slope) and *α* (intercept).	
10:	**for** *k* = 1 to *K* **do**	▹ Isolate phase due to dispersion
11:	$\phi_{k}^{\left(\text{disp}\right)}\leftarrow {\bar{\phi }}_{k}^{\left(\text{M}\right)}-\beta k-\alpha$	
12:	**end for**	
13:	${\bar{f}}_{k}^{\left(\text{S}\right)}\leftarrow \frac{1}{N}\sum_{i=1}^{N}f_{k,i}^{\left(\text{S}\right)}$	▹ Estimate source spectrum
14:	**for** *i* = 1 to *N* **do**	
15:	**for** *k* = 1 to *K* **do**	
16:	$f_{k,i}^{\left(\text{S}\right)}\leftarrow f_{k,i}^{\left(\text{S}\right)}-{\bar{f}}_{k}^{\left(\text{S}\right)}$	▹ Subtract spectrum
17:	${\hat{f}}_{k,i}^{\left(\text{S}\right)}\leftarrow f_{k,i}^{\left(\text{S}\right)}\cdot \text{exp}\left(-i\phi_{k}^{\left(\text{disp}\right)}\right)$	▹ Remove dispersion
18:	**end for**	
19:	**end for**	
20:	**return** ${\hat{f}}_{k,i}^{\left(\text{S}\right)}$	▹ Dispersion-corrected sample fringe data
21:	**end function**	

### Mouse colon imaging

The integrated endoscope was used to image the distal 15mm of colon of a mouse model of colorectal cancer. A/J mice (The Jackson Laboratory, Bar Harbor, ME, USA) were administered the carcinogen azoxymethane (AOM, Sigma-Aldrich, St. Louis, MO, USA) in the cancer group and saline in the control group. Cancer mice were administered 10mg/kg of AOM subcutaneously weekly for five weeks to induce development of colon adenoma, in accordance with a protocol approved by The University of Arizona Institutional Animal Care and Use Committee. Control mice were administered 0.2mL of saline subcutaneously at the same rate. Mice were anesthetized for imaging with intraperitoneal injection of 100mL/kg ketamine and 10mL/kg xylazine. Both mice weighed 20g. Lubrication was applied to the endoscope glass envelope and anus prior to insertion. The endoscope was inserted 30mm into the mouse. Mice were placed on heating pads during imaging to maintain normal body temperature. The mice were not mechanically immobilized during imaging. No maintenance anesthesia was required. The mice recovered after imaging and were monitored for signs of pain or distress.

The 3D OCT volumes consisted of 2400 B-scans and each B-scan consisted of 2000 A-scans. With an A-scan rate of 16 khZ, the B-scan frame rate for 2000 A-scans per B-scan would be 8 frames per second, however the actual frame rate varies due to the variable time for the PC to save the fringe data to disc and is closer to 7.5 frames per second. The rotational velocity of the motor was 2.5rev/s. With a gear ratio of 0.347 linking the rotational motor to the FORJ, this rotational velocity corresponds to an endoscopic rotational velocity of 0.87rev/s. The circumference of the tissue in contact with the glass envelope was about 6.3 mm. At 2000 A-scans samples per B-scan, the lateral sample separation was 3.1 μm. This is approximately half the focused beam radius, therefore the lateral direction is approximately Nyquist sampled. The endoscope moved linearly constantly at a velocity of 41 μm/s. At this speed, imaging of the distal 15mm of the colon took about 6 minutes per mouse. At 2400 B-scans per volume, the sample separation in the longitudinal direction (pitch of the helix) was 6.3 μm, which corresponds to a sampling rate about half the Nyquist criterion.

The adenoma in the AOM-treated mouse were identified by visual inspection of the OCT B-scans using criteria defined by Hariri et al. [4]. These criteria include localized thickening of the mucosa and greater signal attenuation with depth compared to surrounding regions.

## Results and Discussion

**Fig 5:**
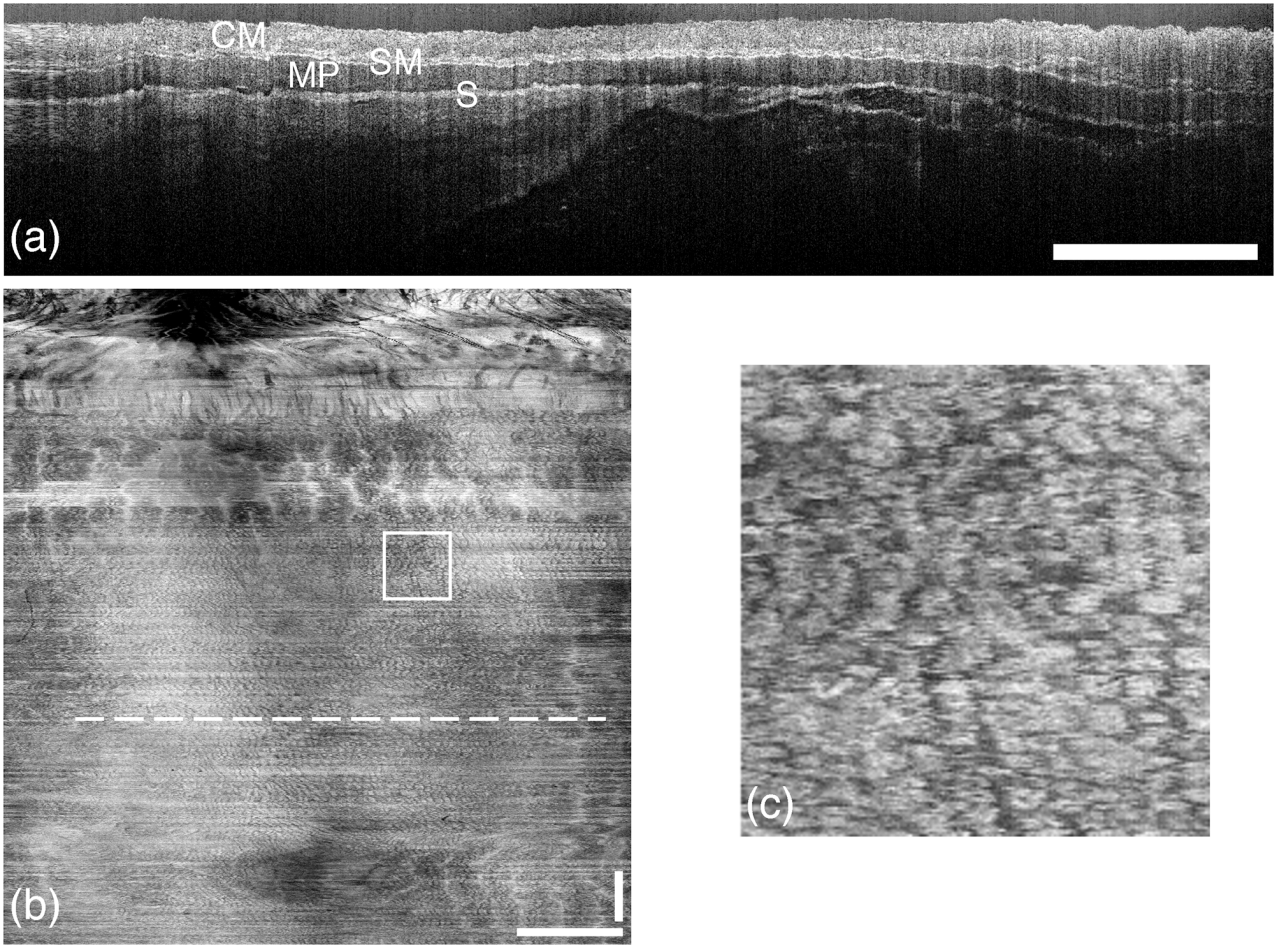
OCT images of saline-treated mouse colon. (a) Cross-sectional image (B-scan) showing high contrast between the many layers of the colon wall (labelled are colonic mucosa (CM), submucosa (SM), muscularis propria (MP), and serosa (S)). The horizontal axis is circumferential and the vertical axis is depth. (b) *En face* standard-deviation projection of the colon with the anus located at the top. The crypt structure can be seen throughout. (c) Enlarged region denoted by the square inset in (b) showing the crypts. The colon circumference (horizontal axis in (a) and (b)) is 6.3mm and the length (vertical axis in (b)) is 15 mm. The horizontal dashed line in (b) indicates the location of the B-scan displayed in (a). All scale bars are 1mm.

**Fig 6:**
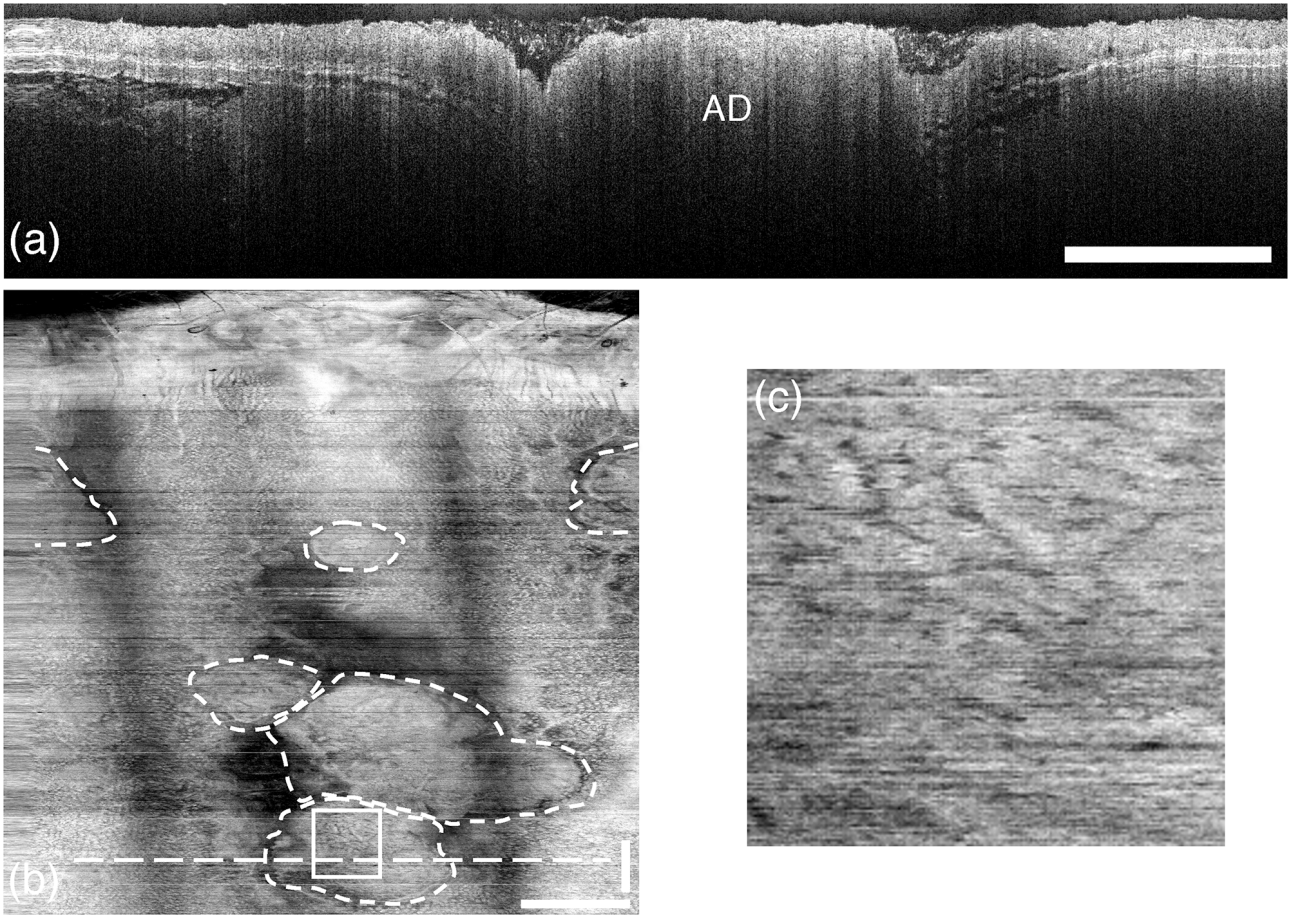
OCT images of AOM-treated mouse colon. (a) Cross-sectional image (B-scan) showing adenoma (AD), characterized by rapid attenuation and loss of tissue boundary visibility. The horizontal axis is circumferential and the vertical axis is depth. (b) *En face* standard-deviation projection of the colon with the anus located at the top. Adenoma are bounded by dashed curves. Crypt structure can also be visualized. (c) Enlarged region denoted by the square inset in (b) showing crypts. Notice the non-uniform crypt density and crypt elongation compared to Fig 5c. The colon circumference (horizontal axis in (a) and (b)) is 6.3mm and the length (vertical axis in (b)) is 15 mm. The horizontal dashed line in (b) indicates the location of the B-scan displayed in (a). All scale bars are 1mm.

We present example OCT images of the distal 15mm of colon for a control mouse (Fig 5) and an AOM mouse (Fig 6). The distortion at the left and right sides of the images are due to oversampling from the acceleration and deceleration of the rotational motor. Both cross-sectional images (Figs 5a and 6a) demonstrate that the system can resolve the epithelium, colonic mucosa, submucosa, muscularis propria, and some tissue beyond the colon wall. The adenoma in Fig 6a appears as a bulbous mass that protrudes from the colonic mucosa.

The *en face* standard-deviation projections in Figs 5b and 6b reveal the colonic crypts (enlarged views in Figs 5c and 6c). The crypts in the healthy mouse are characterized by mostly uniform density and size. By contrast, the crypts on the adenoma in the AOM-treated mouse are elongated and irregular. These results are similar to those previously reported by our group using chromoendoscopy on the same mouse model of colon cancer [27]. Previous endoscopic OCT systems that provided only two-dimensional data visualized murine colonic crypts only intermittently and required ultrahigh resolution [11]. Colonic crypts have been visualized in the larger rabbit using an ultrahigh-resolution light source (200nm bandwidth) [28] or a high-speed (100 kHz) swept-source laser [29]. The projections in Figs 5b and 6b demonstrate that colonic crypts in a mouse colon can be visualized with a relatively simple endoscope and moderately fast commercial swept-source laser system.

Some artifacts are present in the *en face* standard-deviation projections. Respiratory motion and peristalsis of the colon cause axial motion of the tissue. This can cause variability in the overall brightness of a B-scan due to roll off (when the tissue moves away from the endoscope) or reduced contrast due to Doppler shifts.

Compared to our previously reported interim results [16], the images presented here are of higher quality. While the optical design of the endoscope is identical, we corrected some flaws in the construction of the new endoscope by improving the alignment of the optics and reducing the amount of optical glue used cement the components. This improved the power throughput of the endoscope. The axial resolution was also improved through the use of numerical dispersion compensation. The circuit was also redesigned to be more robust and to trigger faster.

Some evidence suggests that aberrant crypt foci may precede dysplasia [30]. Magnifying chromoendoscopy using methylene blue as a contrast agent is the most common method of imaging aberrant crypt foci [31]. The results reported here suggest that OCT may be an attractive contrast-agent-free alternative to chromoendoscopy to investigate the relationship between aberrant crypt foci and cancer development.
